# Effectiveness of a breastfeeding program for mothers returning to work in Japan: a quasi-experimental study

**DOI:** 10.1186/s13006-020-00351-3

**Published:** 2021-01-06

**Authors:** Kaori Nakada

**Affiliations:** Faculty of Nursing, Toho University, 4-16-20, Omori-Nishi Ota-ku, Tokyo, 143-0015 Japan

**Keywords:** Breastfeeding, Mothers, Return to work, Program evaluation, Quasi-experiment design, Japan

## Abstract

**Background:**

Maternal employment has been described as a barrier to breastfeeding in many countries. In Japan, many mothers quit breastfeeding after returning to work because they do not know how to continue breastfeeding. The primary objective of this study was to investigate the effectiveness of a breastfeeding support program for mothers. The secondary objective was to explore the effectiveness of a pamphlet for mothers returning to work.

**Methods:**

This was a quasi-experimental design study with a program group (*n* = 48), pamphlet group (*n* = 46) and comparison group (*n* = 47) that took place from February 2017 to August 2018. Participants in the program and pamphlet groups were women who planned to return to work within 4–12 months after giving birth, while the comparison group included women who had been back at work for at least 3 months. The program involved a 90-min breastfeeding class, a pamphlet, a newsletter, and email consultation. The pamphlet group was sent only the pamphlet, while the comparison group received no intervention. The outcome was breastfeeding continuation rate at 3 months after returning to work.

**Results:**

The breastfeeding continuation rate 3 months after returning to work was significantly higher in the program group than in the comparison group (79.2% vs. 51.1%, *p* = 0.004). After adjusting for background factors, the program intervention had an effect on breastfeeding rates (adjusted odds ratio = 4.68, 95% confidence interval: 1.57, 13.96; *p* = 0.006). However, comparing the pamphlet and comparison groups revealed no significant differences in breastfeeding continuation rates at 3 months after returning to work (69.6% vs. 51.1%, *p* = 0.07).

**Conclusions:**

Program intervention was associated with a significant increase in breastfeeding continuation rates 3 months after returning to work. Randomized controlled trials are needed to make this program applicable in practice. Pamphlet intervention resulted in no significant difference. Further study is necessary after examining the contents of the pamphlet.

## Background

The benefits of breastfeeding for mothers and infants are widely recognized [1, 2]. Nevertheless, early cessation of breastfeeding is common in many developed countries [2, 3]. Maternal employment has been described as a barrier to breastfeeding in numerous studies across many countries and cultures [4]. In addition, the challenge of balancing breastfeeding and employment has been identified as a major barrier to continuation of breastfeeding [2].

Factors affecting working women’s continued breastfeeding include early return to work or timing of return to work, maternal behaviors and characteristics, support at the workplace [2, 5], policy and law [5]. Specifically, returning to work earlier than 6 months postpartum has been identified as a workplace barrier [5, 6]. Type of employment, lower workload [7], shorter working hours [2, 5, 7, 8], increasing maternal age, higher education level [5, 7–9], and mother’s positive attitude towards breastfeeding [5] have been related to longer duration of breastfeeding. Workplace lactation support enhances working mothers’ capacity to continue breastfeeding with employment [5, 10]. A dedicated lactation room, allowance of breast pumping breaks [3, 5, 7], availability of a refrigerator [10], and encouragement from colleagues and supervisors to take breast pumping breaks have been cited as workplace supports [5, 11]. Labor policy on job-protected maternity/paternal leave has the potential to positively influence the duration of exclusive breastfeeding [5, 6, 12]. Work environment strategies targeted at mothers themselves, such as providing options for extended maternity leave and part-time work, and childcare options such as on-site childcare may act as facilitators for breastfeeding continuation [3].

Providing a lactation space and breastfeeding breaks were the two most common breastfeeding supports reported in a previous systematic review [3]. One intervention showed that in person or telephone return-to-work consultations were related to longer duration of breastfeeding [4]. It was suggested that the more support available for mothers, the better the chances of continued breastfeeding [3, 10].

In Japan, many mothers stop breastfeeding shortly after returning to work or before returning to work [13]. This may be because mothers do not know how to continue breastfeeding while working [13]. According to the one study in Japan, after returning to work, the rate of breastfeeding decreases from 44.2 to 7.0% and the rate of infant formula use increases from 7.0 to 46.5% [14]. The reason why breastfeeding does not continue after returning to work is due to the lack of an appropriate workplace and childcare environment [14].

According to one survey, 58.3% of workplaces had a lactation room and 50.0% had a refrigerator to store breast milk [14]. Many daycare centers allow children to drink only artificial milk and do not accept expressed breast milk [14].

The situation of childbirth and return to work in Japan is different from that in other countries. Hospital stay for childbirth is usually 5 days for vaginal delivery. After discharge, if mothers want to receive midwifery support for breastfeeding, they must go to a paid outpatient clinic. Breastfeeding rates have been on the rise for the past 10 years, with 2015 breastfeeding rates reaching 48.4% at 0 months, 51.3% at 1 month, and 53.8% at 6 months [15]. However, in Japan, the term “breastfeeding rate” does not refer to the rate of exclusive breastfeeding. Breastfeeding a baby at the time of the survey is adequate to be considered “breastfeeding”. The number of working women is on the rise, and the number of women who continue working after their first childbirth has increased [16]. In Japan, up to 2 years of childcare leave can be taken after an 8-week maternity leave. According to 2015 statistics, about 35% of women have returned to work within 1 year of giving birth [17]. Breastfeeding support for working women is expected to become increasingly important in the future.

A breastfeeding support program and a pamphlet were developed with information about continuing to breastfeed after returning to work. The primary objective of this study was to investigate the effectiveness of this breastfeeding support program for mothers. The secondary objective was to explore the effectiveness of the pamphlet for mothers returning to work.

The hypothesis of this study was that intervention through the program or pamphlet would increase the rate of breastfeeding 3 months after returning to work compared to no intervention.

## Methods

### Study design

This study used a quasi-experimental design with a program group, pamphlet group and comparison group in an urban area of Japan and was conducted from February 2017 to August 2018. In the program group, the intervention was delivered before returning to work and measured 3 months after returning to work. The program involved a breastfeeding class (90 min), use of a pamphlet, distribution of a newsletter upon returning to work, and email consultation up to 3 months after returning to work. In the pamphlet group, this was sent before returning to work and breastfeeding continuation rate was measured at 3 months after returning to work. The pamphlet was the same one that was used in the program group. No consultation was given to the pamphlet group. Breastfeeding continuation rate was measured in the comparison group only at 3 months after returning to work without intervention. The outcome measure was breastfeeding continuation rate after returning to work.

### Participants and setting

The inclusion criteria of the program group and the pamphlet group were: 1) women who planned to return to work within 4–12 months after giving birth; 2) women who were breastfeeding at the time of recruitment; and 3) women who could communicate and read and write in Japanese. There were no exclusion criteria. The reasons why returning to work after at least 4 months after childbirth was included were as follows. First, breastfeeding rates in Japan rise up to 4 months after birth and are maintained for up to 6 months [18]. Second, since complementary food is started from the age of 6 months, it was thought that women who did not have enough breast milk could continue breastfeeding while using complementary food. To recruit participants for the program group, cooperation was requested from medical and childcare facilities near the program venue. Posters and leaflets requesting participation in the study were distributed at these facilities, and women interested in cooperating were asked to contact the researcher. As a result, program group participants were recruited from seven clinics, four health centers, 19 childcare support centers, five maternity care houses, and four daycares. Prior to the implementation of the program, the purpose of the research was explained verbally and in writing, and consent to participate in the research was obtained. Participants for the pamphlet group were also recruited with the cooperation of medical and childcare facilities. Posters and leaflets requesting participation in the research were distributed at these facilities. As a result, pamphlet group participants were recruited from one hospital, one clinic, nine childcare support centers, five maternity care houses, five daycares, and one private company. The pamphlet was distributed by postal mail, along with a document explaining the purpose of the research, and consent to cooperate was obtained from all participants.

The comparison group included women who returned to work within 4–12 months after giving birth, had been back at work for at least 3 months, were breastfeeding before returning to work, and could read and write in Japanese. There were no exclusion criteria. Comparison group participants were recruited from 22 daycare facilities. A document explaining the research was enclosed with the questionnaire, and returning the questionnaire was interpreted as consent to participate in the research.

In Japan, it is not common for mothers to receive breastfeeding support before returning to work. Therefore, the breastfeeding status of mothers who had already returned to work should reflect the general breastfeeding status.

### Description of the breastfeeding support program

The framework of the program was transformative learning [19], adult learning theory, and empowerment [20]. The program involved a 90-min breastfeeding class, a pamphlet, a newsletter, and email consultation. The purpose of the class was to empower women returning to work by providing knowledge about the continuation of breastfeeding, allowing mothers to discuss breastfeeding with their peers, and for mothers to choose to continue breastfeeding after returning to work. Participants reflect on their breastfeeding experience, recognize the value of breastfeeding, and increase their self-efficacy. This leads to the behavior of choosing to continue breastfeeding after returning to work. At the end of the class, the women wrote an action plan on what to prepare before returning, and how to continue breastfeeding after returning to work.

Adults have a need to be independent in learning, and often realize the need for learning when trying to fulfill developmental tasks and social roles. It is presumed that the participants of this program who are about to return to work have high learning needs. Transformative learning is the process of critically self-reflecting and questioning values. Each participant had experienced breastfeeding since childbirth. After returning to work, when the mothers spent more time separated from their babies, they thought about what they wanted to do with respect to breastfeeding and what was best for the baby. Through small group discussions, mothers were able to share their feelings and worries with each other.

Peer support is effective for breastfeeding support [21–23]. In the class, participants watched a 10-min video presenting the experience of two women who continued breastfeeding after returning to work. One of them fed her child only breast milk, and the other used mixed nutrition. The video showed mothers’ ideas of breastfeeding and the actual conditions before and after returning to work.

The number of participants was limited to 10 people at one time, and group discussions were limited to about five in consideration of group dynamics. Each class was run by two midwives and two support staff with experience in caring for babies. A researcher was in charge of class progress, and another midwife assisted. If there were 10 participants, they were divided into two groups with each midwife facilitating a discussion. If there were fewer than five participants, both midwives participated in the group.

The two support staff took care of the babies and maintained their safety so that the participants could concentrate on the class with confidence. The room environment was arranged so that women and infants could relax together.

Program participants were able to consult with the researcher by email for up to 3 months before returning to work. A newsletter was sent once to the participants before and once after returning to work. The purpose of the newsletter was to share the results of consultations with the participants and prevent them from dropping out of the study. The newsletter was one double-sided, A4-size page printed in color. The contents included bullet-point advice such as preventing problems regarding continuation of breastfeeding.

### Pamphlet structure and contents

The pamphlet contents presented information that could be used before and immediately after returning to work. The pamphlet was in color and consisted of eight, A6-size pages and a cover. The information in the pamphlet included the long-term effects of breastfeeding, how to express breast milk, how to take medications while breastfeeding, weaning, laws related to mothers’ rights in the workplace and the web address of a breastfeeding support organization. In addition, the pamphlet included examples of two women who continued breastfeeding after returning to work. These examples were created based on a previous study [24] that included interviews with 10 women who continued breastfeeding while working and clearly showed preparation and ingenuity to continue breastfeeding, and the actual situation of breastfeeding after returning to work. The contents of the class and the pamphlet were approved by two midwives with extensive breastfeeding experience. The content validity of the pamphlet was reviewed by two midwifery researchers and two women with breastfeeding experience, and was subsequently revised based on their feedback.

### Variables

The outcome was breastfeeding continuation rate at 3 months after returning to work. In this study, breastfeeding continuation was defined as breastfeeding at least once a day. The sample size was calculated assuming that the rate of breastfeeding continuation after returning to work was 60 and 30% in the program and comparison groups, respectively, and that the difference between the two groups was 30%. A power analysis was performed using two-sided analysis with an α error of 0.05 and a power of 0.8. Forty-two participants were needed for each group [25].

The demographic variables were maternal age, month of birth, parity, employment status, education level, smoking status, and previous breastfeeding experience. Breastfeeding-related variables were timing of return to work postpartum, working hours per day, partner’s support in child care, presence of a peer to assist with breastfeeding, consultation with midwifes, and workplace environment for breastfeeding (milk expression breaks, lactation room, refrigerator to store breast milk), and daycare environment for breastfeeding (acceptance of expressed milk). Mothers were also asked about how they were feeding their infant with response choices of breast milk only, infant formula only, and mixed feeding.

### Data analysis

Data were analyzed using descriptive statistics. One-way analysis of variance was used for continuous variables. The chi-square analysis was used for comparison of categorical variables. When the expected frequency was five or less, Fisher’s exact test was performed.

Since it was assumed that background factors of the study participants in each group would influence the intervention outcomes, adjusted results using logistic regression analysis were obtained. The dependent variable was the breastfeeding continuation rate at 3 months after returning to work, and the intervention variable was the program intervention. There were eight independent variables considered to affect the continuation of breastfeeding after returning to work: maternal age, timing of return to work, working hours per day, education level, breastfeeding experience, partner’s support in child care, presence of a peer to assist with breastfeeding, and consultation with midwives. Statistical analyses were conducted using SPSS version 25.0 with a two-sided 5% level of significance.

### Ethical considerations

This study was approved by the Research Ethics Committee of St. Luke’s International University (No. 16-A076) and Kanagawa University of Human Services (No. 10–57). The participants provided written informed consent before study participation.

## Results

### Program participation and questionnaire collection rate

The program was held 12 times with a total of 52 participants. Three months after returning to work, 52 questionnaires were mailed, and 48 were returned (recovery rate, 92.3%). There were 49 participants in the pamphlet group and 48 questionnaires were collected immediately after the intervention. Three months after returning to work, 48 questionnaires were mailed, and 46 were returned (recovery rate, 93.8%). In the comparison group, a total of 123 questionnaires were mailed to 22 facilities, and 67 were returned (response rate, 54.5%). As a result, 47 sets of valid answers (effective response rate, 70.1%) were obtained. There were 48 participants in the program group, 46 participants in the pamphlet group and 47 participants in the control group for a total of 141 included in the final analyses. Missing values included one maternal age in the comparison group. Missing values were included in the analyses as missing without substitution.

### Demographic characteristics

The characteristics of the participants are shown in Table 1. The mean age was 34.0 years [standard deviation (*SD*) = 3.5, *n* = 48] in the program group, 34.8 years (*SD* = 3.9, *n* = 46) in the pamphlet group and 34.2 years (*SD* = 3.9, *n* = 46) in the comparison group, with no significant difference (*p* = 0.58). Parity status (*p* = 0.08) and previous breastfeeding experience (*p* = 0.08) was significantly different among the three groups. Participants in the program group received interventions on average 6.8 months after giving birth (*SD* = 2.3), and the pamphlet group on average 8.2 months after giving birth (*SD* = 2.4).

**Table 1:** Participant characteristics

Variables	Program group (*n* = 48)	Pamphlet group (*n* = 46)	Comparison group (*n* = 47)	*P* value
Maternal age^a^, y (SD)	34.0 (3.5)	34.8 (3.9)	34.2 (3.9)	0.58
Parity status^b^, n (%)				
Primiparous	34 (70.8)	32 (69.6)	24 (51.1)	0.08
Multiparous	14 (29.2)	14 (30.4)	23 (48.9)	
Employment status^b^, n (%)				
Full time	42 (87.5)	36 (78.3)	35 (74.5)	0.26
Part time	6 (12.5)	10 (21.7)	12 (25.5)	
Education level^c^, n (%)				
University or college graguate	47 (97.9)	44 (95.7)	40 (85.1)	0.06
Junior high or high school graduate	1 (2.1)	2 (4.3)	7 (14.9)	
Smoking status^c^, n (%)				
Yes	48 (100.0)	46 (100.0)	45 (97.8)	0.66
No	0 (0.0)	0 (0.0)	1 (2.2)	
No answer			1	
Previous breastfeeding experience^b^, n (%)				
Yes	14 (29.2)	14 (30.4)	23 (48.9)	0.08
No	34 (70.8)	32 (69.6)	24 (51.1)	

*SD* Standard deviation

^a^One-way analysis of variance, ^b^Chi-square test, ^c^Fisher’s exact test

The characteristics of breastfeeding after returning to work are shown in Table 2. The average return to work after giving birth was 9.3 months in the program group (*SD* = 2.6), 9.9 months in the pamphlet group (*SD* = 2.3) and 8.8 months in the comparison group (*SD* = 2.5). The working hours per day were 7.0 (*SD* = 1.1) in the program group, 7.3 (*SD* = 1.2) in the pamphlet group and 6.9 (*SD* = 1.0) in the comparison group. Consultation with midwives before and after returning to work was significantly different between the three groups (*p* = 0.03).

**Table 2:** Participant characteristics of breastfeeding after returning to work

Variables	Program group (*n* = 48)	Pamphlet group (*n* = 46)	Comparison group (*n* = 47)	*P* value
Timing of return to work postpartum (months)^a^, mean (SD)	9.3 (2.6)	9.9 (2.3)	8.8 (2.5)	0.09
Working hours per day^a^, mean (SD)	7.0 (1.1)	7.3 (1.2)	6.9 (1.0)	0.23
Partner’s support in child care^b^				
Yes	45 (93.8)	43 (93.5)	40 (85.1)	0.30
No	3 (6.3)	3 (6.5)	7 (14.9)	
Presence of a peer to assist with breastfeeding^c^, n (%)				
Yes	40 (83.3)	41 (89.1)	37 (78.7)	0.40
No	8 (16.7)	5 (10.9)	10 (21.3)	
Consultation with midwives before and after returning to work^c^, n (%)				
Yes	17 (35.4)	22 (47.8)	10 (21.3)	0.03
No	31 (64.6)	24 (52.2)	37 (78.7)	
Feeding a baby expressed milk at daycare^c^, n (%)				
Yes	22 (45.8)	18 (39.1)	20 (42.6)	0.81
No or unknown	26 (54.2)	28 (60.9)	27 (57.4)	
Lactation room at workplace, n (%)				
Yes	2 (4.2)	3 (6.5)	1 (2.1)	
Secured a lactation room by herself	23 (47.9)	21 (45.7)	10 (21.3)	
No	23 (47.9)	22 (47.8)	35 (74.5)	
Work at home	0 (0.0)	0 (0.0)	1 (2.1)	
Milk expression breaks at workplace, n (%)				
Secured	3 (6.3)	4 (8.7)	3 (6.4)	
Secured break by herself	36 (75.0)	25 (54.3)	13 (27.7)	
Breaks not secured or unknown	9 (18.8)	17 (26.1)	29 (61.7)	
No answer	0 (0.0)	0 (0.0)	2 (4.3)	
Refrigerator to store breast milk, n (%)				
Yes	29 (60.4)	27 (58.7)	12 (25.5)	
No	19 (39.6)	19 (41.3)	31 (66.0)	
No answer	0 (0.0)	0 (0.0)	4 (8.5)	

^a^ One-way analysis of variance, ^b^Fisher’s exact test, ^c^ Chi-square test

The percentage of daycare centres that accepted expressed breast milk was low, at 45.8% in the program group, 39.1% in the pamphlet group and 42.6% in the comparison group. At the workplace, few participants were guaranteed a lactation room and milk expression breaks. Many participants in the program group could make these arrangements by themselves, but the mothers in the comparison group could not. The proportion of participants who had a refrigerator at the workplace to store expressed breast milk was lower in the comparison group (25.5%) than in the program (60.4%) and in the pamphlet (58.7%) groups.

### Outcome

Primary outcomes are shown in Table 3. The breastfeeding continuation rate at 3 months after returning to work was significantly higher in the program group than in the comparison group (79.2% vs. 51.1%, *p* = 0.004). After adjusting for background factors, the program intervention (adjusted odds ratio [AOR] 4.68, 95% confidence interval [CI] 1.57, 13.96; *p* = 0.0060) and maternal age (AOR 1.20, 95% CI 1.02, 1.40; *p* = 0.03) had an effect on breastfeeding rates (Table 4). Secondary outcomes are shown in Table 5. The breastfeeding continuation rates at 3 months after returning to work were not significantly different between the pamphlet group and comparison group (69.6% vs. 51.1%, *p* = 0.07).

**Table 3:** Breastfeeding continuation rate in the program and comparison groups 3 months after returning to work

Variable	Program group (*n* = 48)	Comparison group (*n* = 47)	*P* value
Breastfeeding continuation rate, n (%)			
Yes	38 (79.2)	24 (51.1)	0.004
No	10 (20.8)	23 (48.9)	

Chi-square test

**Table 4:** Logistic regression analysis for breastfeeding continuation in the program group 3 months after returning to work

Variables	*B*	Adjusted odds ratio^a^	95%CI	*P* value
Program intervention				
Yes	1.54	4.68	1.57–13.96	0.006
No				
Maternal age	0.18	1.20	1.02–1.40	0.03
Timing of return to work postpartum	−0.16	0.85	0.69–1.06	0.15
Working hours per day	−0.35	0.70	0.42–1.17	0.70
Education status				
University or college graduate	1.44	4.24	0.58–30.97	0.16
Junior or high school graduate				
Previous breastfeeding experience				
Yes	0.44	1.56	0.49–4.94	0.45
No				
Partner’s support in child care				
Yes	−1.50	0.22	0.05–1.44	0.12
No				
Presence of a peer to assist with breastfeeding				
Yes	−0.01	1.00	0.22–3.93	0.99
No				
Consultation with midwives				
Yes	0.04	1.56	0.46–5.31	0.48
No				

*CI* Confidence interval

^a^Binary logistic regression was adjusted for the variables included in this table

**Table 5:** Breastfeeding continuation rate in the pamphlet and comparison groups 3 months after returning to work

Variable	Pamplet group (*n* = 46)	Comparison group (*n* = 47)	*P* value
Breastfeeding continuation rate, n (%)			
Yes	32 (69.6)	24 (51.1)	0.07
No	14 (30.4)	23 (48.9)	

Chi-square test

In the program group, the mothers could receive consultation with midwives by e-mail. There were eight consultations in six participants (*n* = 48, 12.5%). The consultation topics included methods of cessation breastfeeding at night, methods of disinfecting the breast pump at the workplace, nipple problems, methods of expressing the breast, reduced breast milk production, and how to deal with infants playing with the nipples.

## Discussion

This is the first intervention study on breastfeeding continuation among working women in Japan. The breastfeeding continuation rate at 3 months after returning to work was significantly higher in the program group compared to the comparison group. After adjusting for background factors, the program intervention had an effect on breastfeeding continuation rates and there was an association with breastfeeding continuation 3 months after returning to work.

According to 2017 statistics, the average maternal age at birth of the first child is 30.7 years in Japan, and that of the second child is 32.6 years [26]. Even several months after giving birth, the mothers in the present study were older than the national average. In Japan, the proportion of part time and contract employment is rising and in 2018, 56% of working women were not permanent employees [27]. However, there was a high percentage of full-time workers among the present participants, suggesting that it was relatively easy for them to take maternity and childcare leave. Returning to work 6 months after birth is a factor promoting continuation of breastfeeding [5]. The timing to return to work in this study was 9.3 months in the program group, 9.9 months in the pamphlet group and 8.8 months in the comparison group, suggesting that timing of returning to work was a factor promoting breastfeeding continuation.

Before returning to work, there are specific worries about the condition of the breast and the feeding of the infant that mothers want information and advice about. Other studies have reported that consultation to address these worries before returning to work can be effective in continuing exclusive breastfeeding as well as any breastfeeding after returning to work [4]. In this study, the participants in the program group received intervention on average 6.8 months after giving birth (*SD* = 2.3), and the average return to work after giving birth was 9.3 months (*SD* = 2.6). Therefore, the intervention took place 2–3 months before returning to work. It was speculated that the consultation just before returning to work affected the effectiveness of the program. Face-to-face support has been shown to be more effective than telephone support, and it provides the opportunity to discuss and respond to the mothers’ questions [21]. In this study, individual advice with consideration of the mother’s lactation status and workplace was possible because of face-to-face consultation.

The intervention combined the wisdom of experienced people and the knowledge of professionals. Consultations provided details about the kind of preparations women who continued breastfeeding while working actually made, and when and how they were able to breastfeed and express breast milk while at home, at work, and at daycare. Midwives provided knowledge and information about breast changes after returning to work, and about common problems that mothers face. Before returning to work, it was recommended that mothers check if milk expression and storage could be done at their workplace. The proportion of women who managed to secure a lactation room or milk expression breaks and store their breast milk at the workplace was higher in the program and pamphlet groups than in the comparison group. Participants in the program group knew how to manage breastfeeding continuation after returning to work, which led to them being able to continue breastfeeding.

Returning to work is a major turning point for breastfeeding mothers and children, so it is important to help mothers make choices that they will not regret [23]. If women decide how long and how to continue breastfeeding on their own and make choices they do not regret, they will feel accomplishment in breastfeeding, which will lead to confidence in subsequent childcare.

Expressing breast milk and breastfeeding at the workplace are factors that promote continued breastfeeding after returning to work [28]. In Japan, while long childcare leave is available, women who return to work within 1 year after birth often do not have enough support to continue breastfeeding. There is often no lactation room available at the workplace, so many women express in the rest room, and some women discard expressed milk because there is no refrigerator for storage [24]. A working mother’s decision to continue or discontinue breastfeeding is highly dependent on the support available to her in the workplace [5]. Thus, a comprehensive strategy is required to encourage the practice of breastfeeding in working women from pregnancy to after returning to work [6, 22]. In order for women returning to work in Japan to continue breastfeeding, it is important that employers and daycare centres understand and cooperate with women as they continue to breastfeed. In this study, giving knowledge and information to women led to them being able to continue to breastfeed (behavior change); however, improving the environment of workplaces and daycare centres is an issue that should be addressed immediately.

### Limitations and implications

This study had a selection bias because participants were not randomly assigned to the program, pamphlet and comparison groups. Mothers in the program and pamphlet groups responded to posters requesting participation in the study and volunteered to cooperate, there was the possibility that mothers with a desire to continue breastfeeding were included in the program and pamphlet groups.

There were no statistically significant differences in the demographic data of the participants in the three groups, but there were more multiparous women who had breastfed previously in the comparison group. There were also differences in the work environment after returning to work between the program group and the comparison group which may have affected the results. When assessing the effectiveness of a program, it is necessary to consider that these non-program factors affect outcomes.

### Implications for practice

Program intervention was associated with a significant increase in breastfeeding continuation rates at 3 months after returning to work; however, pamphlet intervention alone resulted in no significant difference. The program was effective but time consuming, and costly due to the personnel needed to run the intervention. It is difficult for women with children to participate in a 90-min program. In the future, it will be necessary to consider a simplified version of the program in consideration of cost and time effectiveness. In addition, with respect to cost-effectiveness, pamphlets are a good tool for providing knowledge and information, and it is important to improve the pamphlet and conduct randomized controlled trials to see the effects. For practical application of the intervention, it is necessary to consider cost performance and media, such as computer and smartphone applications suitable for younger generations who will give birth in the future.

## Conclusions

Program intervention resulted in a significant increase in breastfeeding continuation rates at 3 months after returning to work; and pamphlet intervention alone resulted in no significant differences. Randomized controlled trials are needed to make this program applicable in practice.

## Data Availability

The datasets used and/or analyzed during the current study area available from the corresponding author on reasonable request.

## Abbreviations

AOR: Adjusted odds ratio
CI: Confidence interval
SD: Standard deviation
